# Mineral nutrient remobilization during corolla senescence in ethylene-sensitive and -insensitive flowers

**DOI:** 10.1093/aobpla/plt023

**Published:** 2013-03-28

**Authors:** Michelle L. Jones

**Affiliations:** Department of Horticulture and Crop Science, The Ohio State University, OARDC, 1680 Madison Avenue, Wooster, OH 44691, USA

**Keywords:** Abscission, autophagy, cell death, flowers, nitrogen, petals, petunias, phosphorus

## Abstract

The flower has a finite lifespan that is controlled largely by its role in sexual reproduction. Once the flower has been pollinated or is no longer receptive to pollination, the petals are programmed to senesce. A majority of the genes that are up-regulated during petal senescence, in both ethylene-sensitive and -insensitive flowers, encode proteins involved in the degradation of nucleic acids, proteins, lipids, fatty acids, and cell wall and membrane components. A smaller subset of these genes has a putative role in remobilizing nutrients, and only a few of these have been studied in detail. During senescence, carbohydrates (primarily sucrose) are transported from petals, and the degradation of macromolecules and organelles also allows the plant to salvage mineral nutrients from the petals before cell death. The remobilization of mineral nutrients from a few species has been investigated and will be reviewed in this article. Ethylene's role in nutrient remobilization is discussed by comparing nutrient changes during the senescence of ethylene-sensitive and -insensitive flowers, and by studies in transgenic petunias (*Petunia* × *hybrida*) that are insensitive to ethylene. Gene expression studies indicate that remobilization is a key feature of senescence, but some senescence-associated genes have different expression in leaves and petals. These gene expression patterns, along with differences in the nutrient content of leaves and petals, suggest that there are differences in the mechanisms of cellular degradation and nutrient transport in vegetative and floral organs. Autophagy may be the mechanism for large-scale degradation that allows for recycling during senescence, but it is unclear if this causes cell death. Future research should focus on autophagy and the regulation of ATG genes by ethylene during both leaf and petal senescence. We must identify the mechanisms by which individual mineral nutrients are transported out of senescing corollas in both ethylene-sensitive and -insensitive species.

## Abstract (precis)

The flower has a finite lifespan that is controlled largely by its role in sexual reproduction. The programmed senescence of flowers allows the plant to systematically degrade the petal cells and remobilize nutrients to developing tissues, including the seeds. This senescence program is tightly controlled by the plant hormone ethylene in some flowers, while in some species the senescence signals are unknown. This review article will examine the role of nutrient remobilization during petal senescence and how this differs among flowers with different flower termination phenotypes.

## Introduction

The purpose of the angiosperm flower is sexual reproduction, and some plants have colourful and/or fragrant flowers to aid in attracting pollinators. Despite their importance, it is beneficial for the plant to keep the flowers only as long as they are needed (Ashman and Schoen 1994). The maintenance of petals is costly in terms of respiratory energy, nutrients and water loss (Stead 1992; Stead *et al.* 2006). Once the flower has been pollinated, or the stigma is no longer receptive to pollination, the corolla senesces (Jones 2002). Senescence is the last stage of flower development, during which nutrients are recycled to developing tissues (Bieleski 1995; Verlinden 2003; Chapin and Jones 2007; Trivellini *et al.* 2011). A genetically controlled senescence programme allows the plant to systematically disassemble cellular organelles and macromolecules, and remobilize nutrients from the petals before cell death. The senescence of many flowers is regulated by the plant hormone ethylene (i.e. ethylene-sensitive flowers), while in other plants the initiation and execution of flower senescence is independent of ethylene (i.e. ethylene-insensitive flowers). A flower's lifespan may be as little as a day or up to several months (van Doorn and Woltering 2008).

While a most intriguing aspect of flower senescence is the signalling between the gynoecium and the corolla, most flower senescence research has focused on the biochemical, molecular and morphological changes occurring in the petals, because it is the longevity of the corolla that determines the post-harvest quality and garden performance of ornamental plants (Jones and Woodson 1999). Senescence begins the developmental transition from anabolism of biomolecules to catabolism of lipids, carbohydrates, proteins and nucleic acids (Jones 2004; Rogers 2006; van Doorn and Woltering 2008; Jones *et al.* 2009). Common features of petal senescence include losses of cellular organization, increases in membrane permeability and degradation of organelles (Smith *et al.* 1992; O'Donoghue *et al.* 2002; Wagstaff *et al.* 2003). This is accompanied by the increased export of carbohydrates (especially sucrose), macro- and micro-nutrients; decreased nucleic acid and protein content; increased DNA fragmentation; and increased mRNA abundance and enzyme activity of proteases, nucleases and lipases (Bieleski 1995; Stephenson and Rubinstein 1998; Wagstaff *et al.* 2002; Verlinden 2003; Jones *et al.* 2005; Langston *et al.* 2005; Pak and van Doorn 2005; Yamada *et al.* 2006; Chapin and Jones 2007; Battelli *et al.* 2011). There is increasing ultrastructural evidence and gene expression data indicating that autophagy occurs during petal senescence, but it is unclear if this is required for cell death (Shibuya *et al.* 2011). It does seem likely that autophagy is the mechanism by which macromolecules and organelles are disassembled in senescing petals. This allows for the remobilization or translocation of nutrients from the petals to the developing ovary in a pollinated flower, or to other sink tissues when flowers remain unpollinated.

In this article, I will focus on our current understanding of mineral nutrient remobilization during flower petal senescence. Transcriptome studies using microarrays have identified genes in both ethylene-sensitive and -insensitive flowers that encode catabolic or remobilization proteins, but very little is known about how these genes and their protein products are influenced by pollination, ethylene or environmental stress. The differences in nutrient remobilization during the developmental senescence of unpollinated flowers will be compared with pollinated flowers and with the senescence of short-lived ephemeral flowers. This article will also review critical differences in the senescence of leaves and petals that highlight the importance of conducting thorough comparative studies of these two organs. While many senescence studies have been conducted on cut flowers, an understanding of the biological relevance of nutrient recycling during petal senescence can only come from studies of flowers that are still attached to the plant. Studies of autophagy and transporter proteins (TPs) are increasing our understanding of the mechanisms of cellular degradation and remobilization occurring during senescence, but there is still much to be learned about these processes in flowers.

## Nutrient Remobilization During Petal Senescence

Flower longevity is terminated by either corolla wilting or petal abscission. Corolla wilting is accompanied by a gradual decline in petal fresh weight (FW) and a later decrease in petal dry weight (DW). Petals are visibly wilted when epidermal cells lose turgor. The wilting senescence phenotype occurs in both ethylene-sensitive and -insensitive flowers (van Doorn 2001). In some species, corolla wilting is followed by abscission of petals or the entire flower, while other species shed fully turgid corollas. An intermediate wilting phenotype is observed in some flowers that wilt slightly, concurrent with petal abscission. The decrease in petal DW is due to carbon losses from the respiratory climacteric peak and the export of sugars and mineral nutrients (van Doorn and Woltering 2008).

Autolysis during petal senescence occurs first in the interveinal parenchyma, allowing the phloem to remain viable and export nutrients from the senescing petals. Carbohydrates are mainly transported from the petals as sucrose, and [^14^C]sucrose applied to senescing daylily (*Hemerocallis* sp.) tepals is rapidly translocated to developing flower buds through the phloem (Bieleski 1995). Nitrogen (N) is transported out of the petals as soluble amino acids (Eason *et al.* 2000), and glutamine, hydroxyproline and asparagine are the main transport amino acids identified in the phloem exudates of senescing daylily tepals (Bieleski 1995). Phosphorus (P) is remobilized as the inorganic anion, and many cations, including potassium (K^+^), magnesium (Mg^2+^) and calcium (Ca^2+^), are loaded into the phloem for export during petal senescence (Eason *et al.* 2000; van Doorn and Woltering 2008).

### Changes in petal DW and FW during senescence

In ethylene-insensitive daylily flowers, wilted tepals (petals and sepals) retain only 30–35 % of their maximum DW and <10 % of their FW (Lay Yee *et al.* 1992; Bieleski 1995). Within 24 h of flower opening, ‘Cradle Song’ daylily tepals lose 95 % of their sugar content and 65 % of their DW. Only ∼8 % of this loss can be attributed to respiration and 30 % is accounted for by decreases in non-sugar components (Bieleski 1995). In contrast, some flowers abscise turgid corollas that have lost little to no FW during flower development. Ethylene-sensitive snapdragon (*Antirrhinum majus*) flowers continue to increase petal FW until the fully turgid corolla abscises. This is accompanied by a continual increase in petal DW (Goodwin *et al.* 2003). Flowers like *Alstroemeria* fall somewhere in between, exhibiting slight wilting of both the petals and sepals followed by abscission. Collier (1997) reported that cut *Alstroemeria* (*Alstroemeria pelegrina*) flowers retained only 40 % of their maximum FW, while we have observed less severe wilting in potted *Alstroemeria* (*Alstroemeria hybrid* ‘Ivana’) plants, with flowers still attached to the plant retaining 75–80 % of their FW. Dry weight losses were similar in both the cut flowers and attached flowers, with newly abscised petals retaining 80 % of their maximum DW (Collier 1997; M. L. Jones, unpubl. data).

### Macro- and micro-nutrient changes in petals during the developmental senescence of unpollinated flowers

Experiments evaluating the macro- and micro-nutrient content of the corolla from anthesis (flower opening) through senescence provide evidence for the remobilization of individual mineral nutrients. A number of experiments evaluating nutrient changes during flower development have been conducted in *Petunia* × *hybrida* (Verlinden 2003; Chapin and Jones 2007, 2009). Petunia is often used as a model for ethylene-sensitive flower senescence (Jones *et al.* 2009). Senescing, unpollinated petunia flowers show visual symptoms of corolla wilting, concomitant with decreases in FW at 6–10 days after anthesis, depending on growing conditions (Verlinden 2003). Decreases in petunia petal DW during senescence are not as extensive as those in daylily, and they retain ∼65 % of their maximum DW (Verlinden 2003).

The natural senescence of unpollinated ‘Mitchell Diploid’ (MD) petunia flowers (a.k.a. developmental or age-related senescence) is accompanied by decreases in the N and P content of the corollas (Verlinden 2003; Chapin and Jones 2009). A gradual decline in P throughout development begins well before visual symptoms of wilting or losses in FW. In contrast, the N content of the corolla starts to decline during the later stages of senescence when the petals are wilted and producing ethylene. The P content of unpollinated senescing corollas decreases by 74–75 %, and N decreases by 50–60 % (Verlinden 2003; Chapin and Jones 2009). While a larger percentage of the total P appears to be recovered during the senescence of petunia corollas, this amounts to only 92 µg of P on average per corolla. The N decrease from the corolla averages 390 µg. This loss in P and N represents only 4 and 17 %, respectively, of the total decrease in DW that occurs during the senescence of unpollinated corollas (Chapin and Jones 2009).

It is interesting to note that the amount of P remobilization reported in experiments conducted independently by the Verlinden (West Virginia University) and Jones (The Ohio State University) labs are very similar (74 and 75 % remobilization), while N changes were more variable. Verlinden (2003) also reports a steady decline in the K content of MD petals during the senescence of unpollinated flowers (∼40 %), while we have consistently observed that the K content of the corolla increases slightly during development, with final levels in the fully senescent (late-stage senescing) corollas greater than, or the same as, those in corollas at anthesis (Chapin and Jones 2009). In contrast, we have measured a decrease in the K content of ∼35 % in senescing corollas from pollinated flowers (Chapin and Jones 2007; see below for more about differences between the senescence of pollinated and unpollinated flowers). These differences may suggest that the remobilization of N and K are influenced more by environmental conditions and the nutritional status of the plant than P. All other macro- and micro-nutrients evaluated either increased in abundance during senescence or were the same when comparing corollas from fully senescent flowers to those at anthesis (Verlinden 2003).

### Macro- and micro-nutrient profiles differ during pollination-induced and developmental senescence

**Figure 1.**
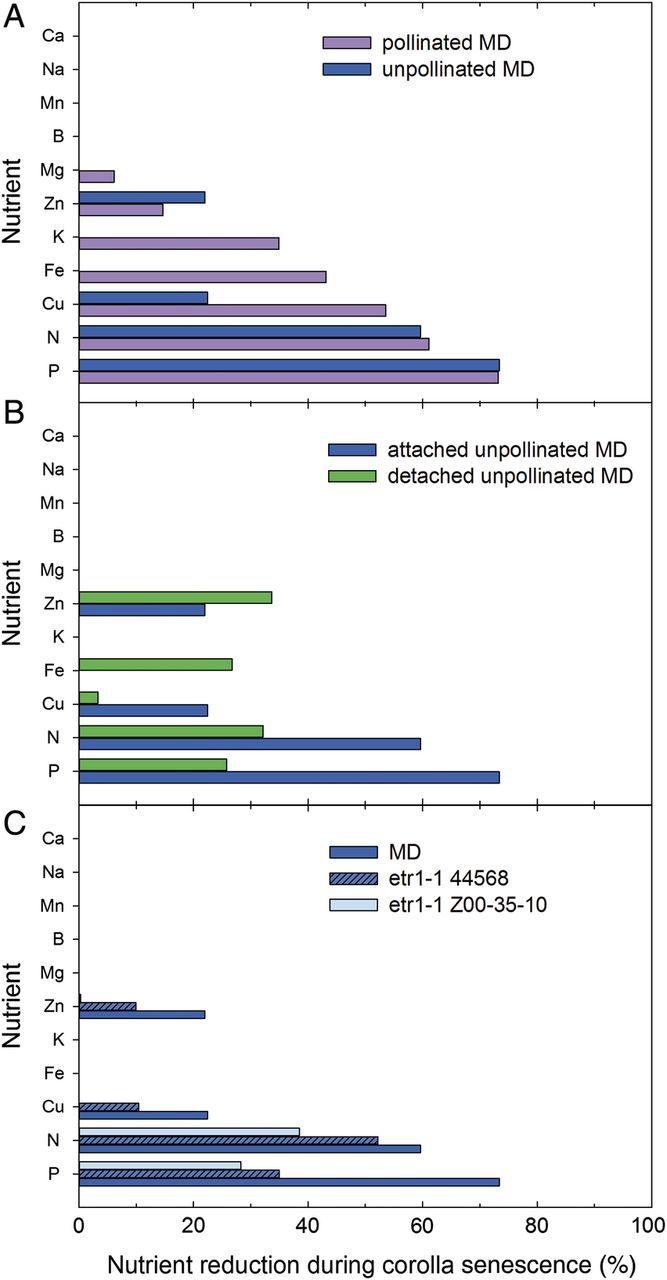
Summary of the percent reduction in macro-nutrients and micro-nutrients during *Petunia* × *hybrida* corolla senescence. The total nutrient content of corollas at anthesis was compared with nutrient content at the advanced stage of senescence (fully wilted) as an indication of nutrient remobilization. (A) Nutrient remobilization in MD petunia corollas during pollination-induced senescence and during the developmental senescence of unpollinated corollas. (B) Nutrient remobilization in MD corollas during the senescence of unpollinated flowers attached to the plant or in detached, cut flowers. (C) Nutrient remobilization in MD wild-type corollas and transgenic corollas (lines Z00-35-10 and 44568) with reduced sensitivity to ethylene due to the expression of the mutated ethylene receptor (35S::*Atetr1-1*). Line 44568 *etr1-1* is less sensitive to ethylene than line Z00-35-10 *etr1-1*.

Pollination accelerates the senescence of many longer-lived flowers (including petunia), and this response is dependent on ethylene signalling (Jones 2008). When petunias are pollinated, the corollas wilt within 48 h (Chapin and Jones 2007). During developmental senescence, wilting of unpollinated petunias occurs at 6–10 days after anthesis. While only the macronutrients N and P (and in some instances K) are remobilized during the senescence of unpollinated flowers (i.e. developmental senescence), additional nutrients are remobilized during pollination-induced senescence in petunia flowers. Nitrogen and P are reduced to a similar extent in both unpollinated and pollinated corollas (Fig. 1A; Verlinden 2003; Chapin and Jones 2007, 2009). In addition, as mentioned previously, the senescent corollas from pollinated flowers have a 35 % reduction in the K content. The macro-nutrient Mg also shows a small, but significant decrease of ∼6 %. The micro-nutrients zinc (Zn) and copper (Cu) decrease during pollination-induced senescence, with the largest decreases in Cu content (54 % reduction).

In pollinated petunias (detached flowers), N increases in the ovary were found to occur concomitantly with decreases in petal N content (Shibuya *et al.* 2013), suggesting that the nutrients that are remobilized from pollinated senescing corollas are transported to the developing ovary. Unfortunately, N was the only nutrient measured in this study. The post-pollination response in orchids is also associated with increased ethylene production and accelerated wilting of the perianth (calyx plus corolla). Pollination results in an increase in the FW and DW of the gynostemium and the ovary, and a decrease in the FW and DW of the perianth (Hew *et al.* 1989). These changes have been associated with the transport of N and phosphate from the perianth to the ovary in *Cymbidium* and *Arachnis* orchids (Harrison and Arditti 1976; Hew *et al.* 1989). The movement of these nutrients is dictated by sink activities. When radiolabelled P is applied to the labella (lips and median petals) of unpollinated *Cymbidium* flowers, ^32^P is detected throughout the flower, with the lowest levels in the perianth. Pollination redirects this transport, and the largest accumulation of ^32^P is detected in the gynostemia (column) and the ovaries (Harrison and Arditti 1976). The N and P gains in the ovary of *Arachnis* orchids are much greater than the losses from the perianth, suggesting that ovule development also requires the additional remobilization of nutrients from sources outside the flower (Hew *et al.* 1989).

It has been proposed that pollination-induced ethylene production in the orchid flower regulates the changes in sink activity of the ovary that drive nutrient transport (Hew *et al.* 1989). Treating carnation flowers with exogenous ethylene mimics pollination, accelerating the wilting of the petals and increasing the growth of the ovary (Nichols 1976). In this experiment, cut flowers were treated with 1 µl L^−1^ ethylene for 1 day. After 4 days, the DW of the petals decreases by 29 % and this is accompanied by a decrease in N (43 %), P (53 %) and K (27 %). Ovary DW increases and the N, P and K content of the ovary also increases (Nichols 1976). These experiments provide support for ethylene's role in remobilization of nutrients from the petals to the developing ovary.

Transporting nutrients within the plant requires energy, so the specific nutrient and the amount of each nutrient that is remobilized must result in a net advantage to plant growth and reproductive development. The largest remobilization from corollas occurs with N and P, which are the most likely to limit plant growth, and would therefore be the most advantageous for the plant to recycle. It takes an additional investment of energy and resources to remobilize nutrients outside the flower (when flowers remain unpollinated) versus within the flower (following pollination or ethylene treatment). This additional ‘cost’ of recycling nutrients from unpollinated flowers may explain the differences in the remobilization profiles observed during developmental and pollination-induced senescence in longer-lived flowers. Additional experiments with ^32^P are needed to track the movement of nutrients from the petals to the developing ovary, and to determine when nutrients are remobilized to alternative sinks.

### Nutrient remobilization during the senescence of ephemeral flowers

Perhaps more surprising is the fact that the very rapid senescence programme of ephemeral flowers also involves a nutrient remobilization phase. The changes in the nutrient profiles of senescing *Hibiscus rosa-sinensis* (hibiscus) are very similar to the pollination-induced changes in petunia corollas (Chapin and Jones 2007; Trivellini *et al.* 2011). Hibiscus is an ethylene-sensitive, ephemeral flower that opens and wilts within 1 day. This very rapid senescence programme is accompanied by decreases in P, N, Cu, K, Zn and Mg, the exact same nutrients that are remobilized during the accelerated senescence of pollinated petunia corollas. In this experiment, hibiscus plants were grown in the greenhouse. There was no mention of hand pollination, and the greenhouse environment would reduce the chance of pollination by birds or insects. The lack of an increase in the DW of ovaries from the open to senescing flower stages also supports the assumption that these flowers were not pollinated. Similarly, the senescence of ethylene-sensitive *Ipomoea purpurea* (morning glory) flowers is accompanied by a 75 % reduction in the P content of the petals, and a 65, 55 and 34 % reduction in the amount of K, Mg and Ca, respectively (Winkenbach 1970). The senescence of ephemeral flowers is generally not accelerated by pollination. Nutrient remobilization studies indicate that the senescence of these flowers may be more similar to pollination-induced senescence than developmental senescence in their longer-lived counterparts (Winkenbach 1970; Trivellini *et al.* 2011). In Japanese morning glories, petal senescence starts before the flowers are even open (Winkenbach 1970). This early initiation of senescence would be needed to allow for cellular degradation and nutrient remobilization in such short-lived flowers.

### Senescence of cut flowers versus flowers that are attached to the plant

Many studies of flower senescence have been conducted in cut flowers that have been harvested from the plant and held in water or a vase solution (Wagstaff *et al.* 2003; Breeze *et al.* 2004; Pak and van Doorn 2005; Hoeberichts *et al.* 2007; Battelli *et al.* 2011). While excellent studies of senescence have been conducted in these systems, we must interpret these results with caution when it comes to the nutrient remobilization phase of senescence. In a cut stem with a single flower, like standard carnations (*Dianthus caryophyllus*), nutrient remobilization from petals of unpollinated flowers may be reduced compared with flowers that are still attached to the plant, if nutrient translocation is driven by sinks outside of that flowering stem. In contrast, inflorescence or spike-type cut flowers, which have multiple flowers at different developmental stages, may provide an excellent model for studies of remobilization from senescing to developing flowers. Removal of the lower three open flowers from a *Gladiolus* spike reduces the total DW of newly opened florets, indicating that the older flowers on the spike are an important source of nutrients for the newly developing flowers (Waithaka *et al.* 2001).

To investigate nutrient remobilization in a cut flower system, detached, single petunia flowers were placed in a vial of distilled water and allowed to senesce. The nutrient content of corollas at anthesis (on the day of flower opening from a flower just harvested from the plant) was compared with the nutrient content of corollas from detached flowers that had been allowed to senesce off the plant (Fig. 1B; M. L. Jones, unpubl. data). In the detached flowers, the N and P content of the corollas decreases during senescence, but remobilization is reduced compared with corollas from attached flowers that senesced on the plant. The reduction in N is only 32 % and P is 26 %, compared with 60 and 74 % in attached flowers. The only other difference between attached and detached flowers is a decrease in the iron (Fe) content (27 %) of senescing detached corollas that is not detected in attached corollas. The senescence of detached daylily flowers is visually similar to the senescence of attached flowers, but there are major differences in tepal composition. Senescing tepals from detached flowers have a higher FW, DW, sugar and amino acid content than tepals from attached flowers (Bieleski 1995). These studies show that nutrient remobilization from the petals is reduced when flowers are detached from the plant during senescence. Further studies are needed to determine the signals that regulate nutrient remobilization, but it is clear that studies of developmental senescence should include flowers that are still attached to the plant.

### Ethylene regulation of nutrient remobilization during petal senescence

Macro- and micro-nutrient changes have also been investigated in transgenic petunias that have reduced sensitivity to ethylene due to ectopic expression of the mutated ethylene receptor *etr1-1* (35S::*Atetr1-1*; Wilkinson *et al.* 1997). These plants serve as an excellent model system for investigating the role of ethylene in nutrient remobilization (Chapin and Jones 2009). Two different transgenic lines, Z00-35-10 and 44568 (obtained from David Clark, University of Florida), were used for these studies. Both lines have extremely reduced responsiveness to ethylene, but line 44568 is less sensitive than Z00-35-10.

In *etr1-1* flowers, the N content is reduced by 52 and 38 % during the senescence of unpollinated corollas in the Z00-35-10 and 44568 lines, respectively (Fig. 1C; Chapin and Jones 2009). This is compared with a 60 % reduction in the MD flowers. While ethylene signalling may influence the transport of N out of the senescing petals, the influence of ethylene on P remobilization during senescence is much more striking. While 73 % of the P is remobilized from MD corollas, only 35 and 28 % of the P is remobilized from the *etr1-1* Z00-35-10 and 44568 lines, respectively. The final seed weight of *etr1-1* petunias is also significantly different from that of MD flowers, which may be in part due to reduced N and P remobilization to the ovary (Clevenger *et al.* 2004).

### The involvement of TPs in nutrient remobilization within the flower

Transporter proteins are critically involved in the process of nutrient remobilization. Large-scale expression profiling in leaves has identified TPs from many different classes that are up-regulated during the senescence of Arabidopsis (*Arabidopsis thaliana*) leaves (van der Graaff *et al.* 2006), but fewer TPs have been identified in petals. Even fewer have been characterized to any extent to investigate their role in petal senescence, or to determine how they are regulated by ethylene. Microarray studies in *Alstroemeria* and carnation petals have identified senescence up-regulated genes that are classified as transporters (Breeze *et al.* 2004; Hoeberichts *et al.* 2007). In carnation petals, a metal–nicotianamine (NA) transporter (Hoeberichts *et al.* 2007) is up-regulated during petal senescence. These NA transporters belong to the oligopeptide transporter family and have been implicated in metal transport in plants. Also called yellow stripe-like (YSL) proteins in rice, they are involved in metal detoxification and specifically transport iron and manganese (Mn)–NA chelates in the phloem (Ishimaru *et al.* 2010; Sasaki *et al.* 2011). The role of YSL proteins in petals warrants further investigation, and may shed some light on the role of metal transport in and out of the flower during development and senescence.

Five high-affinity phosphate transporter genes have been identified in petunia (Wegmuller *et al.* 2008; Chapin and Jones 2009). The majority of these are expressed in roots and believed to be involved in Pi uptake. *PhPT1* transcripts are detectable at low levels in non-senescing petals and increase during senescence (Chapin and Jones 2009). The highest transcript abundance correlates with the large decreases in P content that accompany corolla senescence. *PhPT1* transcript abundance is reduced in both Z00-35-10 and 44568 *etr1-1* petunias, supporting the aforementioned conclusion that P remobilization involves ethylene signalling. Treating flowers with a very low concentration of ethylene (0.1 µl L^−1^) for only 2 h results in up-regulation of *PhPT1* transcripts (Chapin and Jones 2009). Treatment with cycloheximide does not prevent this induction, indicating that *de novo* protein synthesis is not required for this response, and therefore *PhPT1* is an ethylene primary response gene. This is unusual for a gene involved in the nutrient remobilization phase of senescence. Most of the senescence-associated genes, including *SAG12* in Arabidopsis, are up-regulated by ethylene as a secondary response (Weaver *et al.* 1998). This can explain why P levels start to decrease in the petals earlier than N. The primary regulation of *PhPT1* by ethylene may also allow the plant to use the P in the corolla as a reserve during P starvation or other stresses that induce senescence.

A putative nitrate transporter (*DAFSAG7*) is up-regulated during the senescence of *Narcissus pseudonarcissus* (daffodil) tepals. Transcripts are not detected in the perianth of newly open flowers, but are first detected at 3 days after opening, just prior to tepal discolouration. Transcript abundance increases during perianth senescence, supporting a role for this nitrate transporter in the nutrient remobilization phase of flower senescence (Hunter *et al.* 2002). A putative peptide transporter (*WC11*) is up-regulated in both *Erysimum linifolium* (wallflower) petals and leaves (though to a greater extent in petals) (Price *et al.* 2008). *WC11* has a much higher basal level in non-senescing leaves. It is up-regulated early in the senescence programme and decreases by the time that leaves are visually yellowing and *SAG12* (often used as a marker for senescence) is up-regulated. The *WC11* transcripts are induced to a greater extent early in petals, well before visual senescence, and remain high throughout senescence (Price *et al.* 2008). The closest Arabidopsis homologue to *WC11* is At1g32450, which encodes a xylem nitrate-loading transporter that is involved in nitrate allocation to roots and responses to drought, salinity and cadmium stress (Chen *et al.* 2012). This expression pattern does not match what we would have expected based on the late N export patterns from petunia corollas, but may suggest that this transporter also has a role in N transport into young sink tissues (either leaves or petals).

## Autophagy

Recent research has suggested that autophagy is the mechanism for the degradation of macromolecules during the senescence of flower petals (Yamada *et al.* 2009). Autophagy is a type of programmed cell death first identified in animal cells that is characterized by the appearance of autophagosomes, vacuole-like vesicles involved in degradation of the cytosol (van Doorn 2011). This process is also defined by the activation of autophagy or ATG genes. Homologues to the Arabidopsis autophagy-related proteins *ATG4b* and *ATG8a* have been identified in *Ipomoea nil* (Japanese morning glory) petals (Yamada *et al.* 2009; Shibuya *et al.* 2011). Morning glories are ethylene-sensitive, ephemeral flowers that open and senesce in 1 day, and the senescence programme in mesophyll cells is initiated even prior to flower opening. Petal senescence is accompanied by an increase in transcript abundance for both *InATG4b* and *InATG8a*. Ethylene treatments that accelerate flower senescence up-regulate *InATG4b* but not *InATG8a* expression (Yamada *et al.* 2009). The presence of autophagosome-like vesicles has also been demonstrated in the cytoplasm and the vacuole (Matile and Winkenbach 1971; Matile 1997). Monodansylcadaverine, which has been shown to stain autophagosomes, has identified the appearance of autophagic structures during petal senescence in both morning glory and petunia (Shibuya *et al.* 2009, 2013). In petunia flowers, pollination-induced and developmental senescence is accompanied by an increase in transcript abundance for *PhATG8* homologues (ATG8a, ATG8b, ATG8c and ATG8d) (Shibuya *et al.* 2013). Additional research is needed to determine the differential regulation of ATG genes by ethylene. Functional analysis of the ATG genes in petunia using RNAi or virus-induced gene silencing should provide more evidence for or against the role of autophagy in developmental and pollination-induced petal senescence.

## Differences Between Leaf and Petal Senescence

Few studies of leaf and petal senescence have been conducted in the same species, as the ideal model systems for petal senescence, including petunia, do not have distinct and predictable leaf senescence like Arabidopsis. It is also difficult to identify physiological or morphological characteristics that can be used as developmental benchmarks to ensure that equivalent senescence stages are being compared between leaves and petals. Comparative analyses of gene expression in Arabidopsis (Wagstaff *et al.* 2009) and wallflower (Price *et al.* 2008), a relative of Arabidopsis that has large ornamental flowers, have identified key metabolic pathways that are shared between senescing leaves and petals, as well as genes that are up-regulated only in petals. These studies have identified senescence pathways that are tissue specific and which validate the need to study senescence in both petals and leaves.

Genes associated with autophagy, degradation and remobilization are up-regulated in both leaves and petals of Arabidopsis and wallflower, but differences in the expression profiles of specific genes are apparent (Price *et al.* 2008; Wagstaff *et al.* 2009). Within the *APG8*/*ATG8* subfamily in Arabidopsis, members *APG8a* and *APG8b* are up-regulated during both petal and leaf senescence. In contrast, *APG8f* and *APG8i* are up-regulated during petal senescence, but earlier in leaf development (at 16 days after emergence). *APG8e* shows little change during leaf development and senescence, but is up-regulated during petal senescence (Wagstaff *et al.* 2009). These findings suggest that differences in cellular constituents and nutrient content have led to the differences in autophagy that allow for the specific mechanisms of degradation and nutrient remobilization in senescing leaves and petals. In wallflower, the identification of genes encoding proteins involved in degradation and remobilization (e.g. a copper chaperone, lipid transfer protein, thiol protease and AAA-type ATPase family protein), which are up-regulated during petal senescence and not during leaf senescence, provides additional evidence for differences in the remobilization phase of senescence in petals versus leaves (Price *et al.* 2008).

Studies in Arabidopsis indicate that 80 % or greater of the N, P and K content is remobilized from senescing leaves. In addition to the Cu, Zn and Mg that are remobilized in senescing (pollinated) petunia and hibiscus petals, levels of chromium (Cr), Fe and molybdenum (Mo) also drop by >40 % during Arabidopsis leaf senescence. Smaller reductions (∼15 %) are observed in the cobalt (Co), nickel (Ni), Mn, sodium (Na) and Ca content (Himelblau and Amasino 2001). In the comparative study of leaf and petal senescence in wallflower, the extent of protein degradation was found to be much greater during the senescence of leaves. The senescent leaves (stage 7) retain only 5 % of maximum protein levels compared with 65 % in senescent petals (stage 5) (Price *et al.* 2008). This decrease in protein content is concomitant with chlorophyll degradation, and is not surprising considering that the majority of the remobilization occurring in leaves is due to the degradation of chloroplast proteins (Thomas and Donnison 2000).

**Figure 2.**
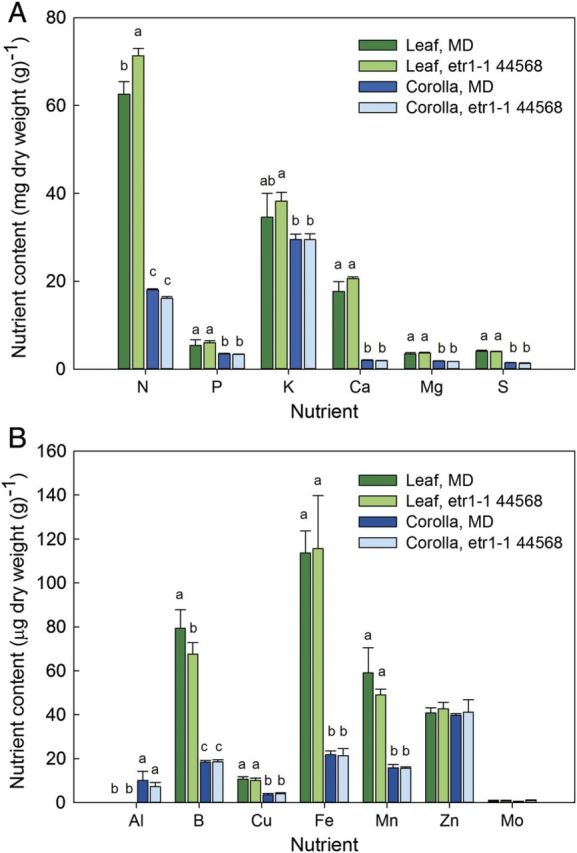
Nutrient content of leaves and corollas from *Petunia* × *hybrida*. The (A) macro-nutrient and (B) micro-nutrient content of corollas from flowers at anthesis was compared with the nutrient content of young, newly expanded green leaves. Plants included wild-type MD and transgenic *etr1-1* (44568) petunias with reduced sensitivity to ethylene.

Petals are fairly nutrient poor compared with leaves, and these experiments indicate that leaves remobilize a greater total amount and larger number of individual nutrients during senescence (Himelblau and Amasino 2001; Verlinden 2003; Chapin and Jones 2007, 2009). When comparing young, newly expanded green petunia leaves to petunia corollas on the day of anthesis (Fig. 2), the largest difference is the N content. Leaves contain 63 mg N per g DW, while petals contain only 18 mg N per g DW. That is over 300 % more N in leaves than in petals. The difference between the P and K content of leaves and petals is much smaller. Of the other macro-nutrients, the largest difference between leaves and petals is seen with Ca. Among the micro-nutrients, the greatest differences between leaves and petals are in B, Fe and Mn content. The nutrient content of *etr1-1* petals is similar to that of MD petals, indicating that ethylene signalling is not required for the accumulation of nutrients in non-senescing, developing corollas. The nutrient content of young leaves is also similar, with the exception of N, which is consistently higher in *etr1-1* leaves, and B, which is higher in MD leaves (Fig. 2).

Interestingly, the micro-nutrients that are remobilized from pollinated petals, Cu and Zn, are also nutrients that have the same or similar concentrations in leaves and petals. If specific nutrients are much higher in the leaves than in the petals, plants may invest in recycling them only from the more nutrient-rich tissues. If specific nutrients are needed as cofactors or activators for senescence-related enzymes, this may explain why these nutrients continue to increase in abundance or remain at similar levels, rather than being remobilized out of the petals during senescence. For example, Fe is at a much lower concentration in petals than leaves, and it is retained in the petals during flower senescence. Iron is a cofactor for the enzyme ACC oxidase, which catalyses the autocatalytic burst of ethylene that is synthesized by the petals during senescence (Zhang *et al.* 1995). The petals may need to retain Fe during senescence to allow for ethylene production.

## Conclusions

Flowers may abscise fully turgid corollas or petals may be severely wilted before they are eventually shed. There are also intermediate wilting phenotypes that fall in between these two extremes. The wilting of flowers is accompanied by remobilization of mineral nutrients (primarily N and P) from the petals. This nutrient remobilization has been demonstrated in ethylene-sensitive flowers (e.g. petunia, hibiscus, morning glory and orchids) and ethylene-insensitive flowers (e.g. daylily). Ethylene appears to regulate some components of the remobilization pathways in ethylene-sensitive flowers, as is seen with P transport out of senescing petunia petals and expression of the phosphate transporter *PhPT1* (Chapin and Jones 2009). *Alstroemeria* flowers, which senesce and abscise independently of ethylene action, show an intermediate wilting phenotype. While gene expression changes indicate up-regulation of proteases and other genes encoding proteins involved in cellular degradation (Wagstaff *et al.* 2002; Breeze *et al.* 2004), it is not clear how much of the senescence programme is completed in these flowers before the petals and sepals are shed. It can be assumed that senescence is not occurring in petals from flowers (e.g. digitalis and snapdragon) that abscise fully turgid corollas, but there are no gene expression or nutrient remobilization studies to support or refute this claim. Nutrient changes must be investigated in abscission type, and more importantly, in intermediate wilting type flowers, to provide us with a more complete understanding of the role that nutrient remobilization from the petals plays in the terminal phases of flower development.

Increasing evidence suggests that autophagy is involved in petal senescence, but studies are needed to characterize the expression of individual ATG genes in petals from multiple species, and to determine if this process is regulated by ethylene. It is not clear if autophagy is the cause of cell death, or if it is a mechanism that allows for efficient remobilization by delaying cell death until nutrients can be recycled. While no one seems to disagree that nutrient recycling is the central function of senescence in both leaves and petals, it is less clear if and when mineral nutrient remobilization from petals will have an important impact on the overall fitness or reproductive success of the plant. I have postulated that salvaging N or P, nutrients that are most likely to limit plant growth, from the petals may have a positive impact on the overall nutrient status of the plant. While this may be true, it may be under nutrient starvation conditions where recycling mineral nutrients from the petals is most important. The nutrients that can be remobilized from the corolla to the ovary and developing seeds may be increasingly important under these conditions and may allow for successful reproduction under environmental stress. This is an area of flower senescence that has received little attention and must be addressed to truly understand the biological implications of petal senescence.

Comparative transcriptome analyses between flowers with different senescence syndromes have been limited by the lack of sequence data in all but a few model species. The increased accessibility and decreased cost of next-generation sequencing should result in a large increase in sequence availability, and allow for more comprehensive investigations of the petal senescence transcriptomes in ethylene-sensitive and - insensitive species. These experiments will also allow us to identify the extent of the senescence programme that occurs in species like *Alstroemeria*, which show limited wilting before the petals are shed. These studies, in combination with proteomic (Bai *et al.* 2010) and metabolomic investigations, will help us to further our understanding of the biological relevance of nutrient remobilization during petal senescence, and allow us to determine how petal senescence is regulated by ethylene, during development and under stress.

## Sources of Funding

The author's research was funded by The Ohio State University D.C. Kiplinger Floriculture Endowment, the American Floral Endowment, The Fred C. Gloeckner Foundation, and the United States Department of Agriculture Floriculture and Nursery Research Initiative. Salaries and research support were provided in part by State and Federal funds appropriated to the Ohio Agricultural Research and Development Center, The Ohio State University.

## Conflict of Interest Statement

None declared.
